# Supplementary figures and images for: Growth, stoichiometry and cell size; temperature and nutrient responses in haptophytes
Source: PeerJ. 2017 Sep 5;5:e3743. doi: 10.7717/peerj.3743 (PMC5590550; doi:10.7717/peerj.3743)

Alkaline phosphatase activity (relative units)

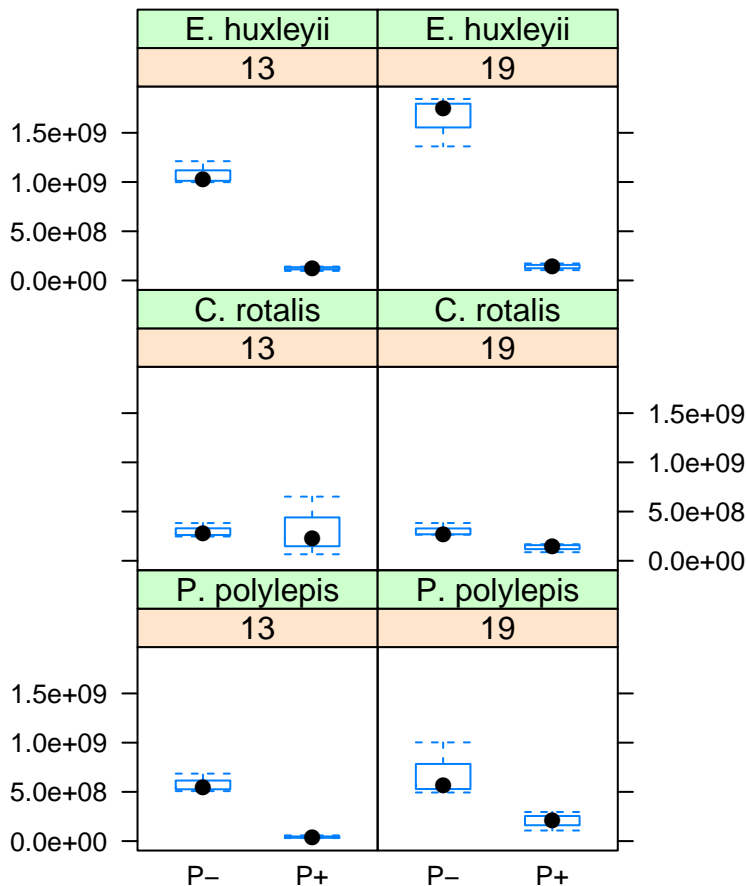

Supplement: Figure S1 — Alkaline phosphatase activity (APA) plotted as a function of P-regime (P + is nutrient replete cultures, P − is P-limited cultures) given temperature (13 °C and 19 °C) and species. [file peerj-05-3743-s002.pdf]

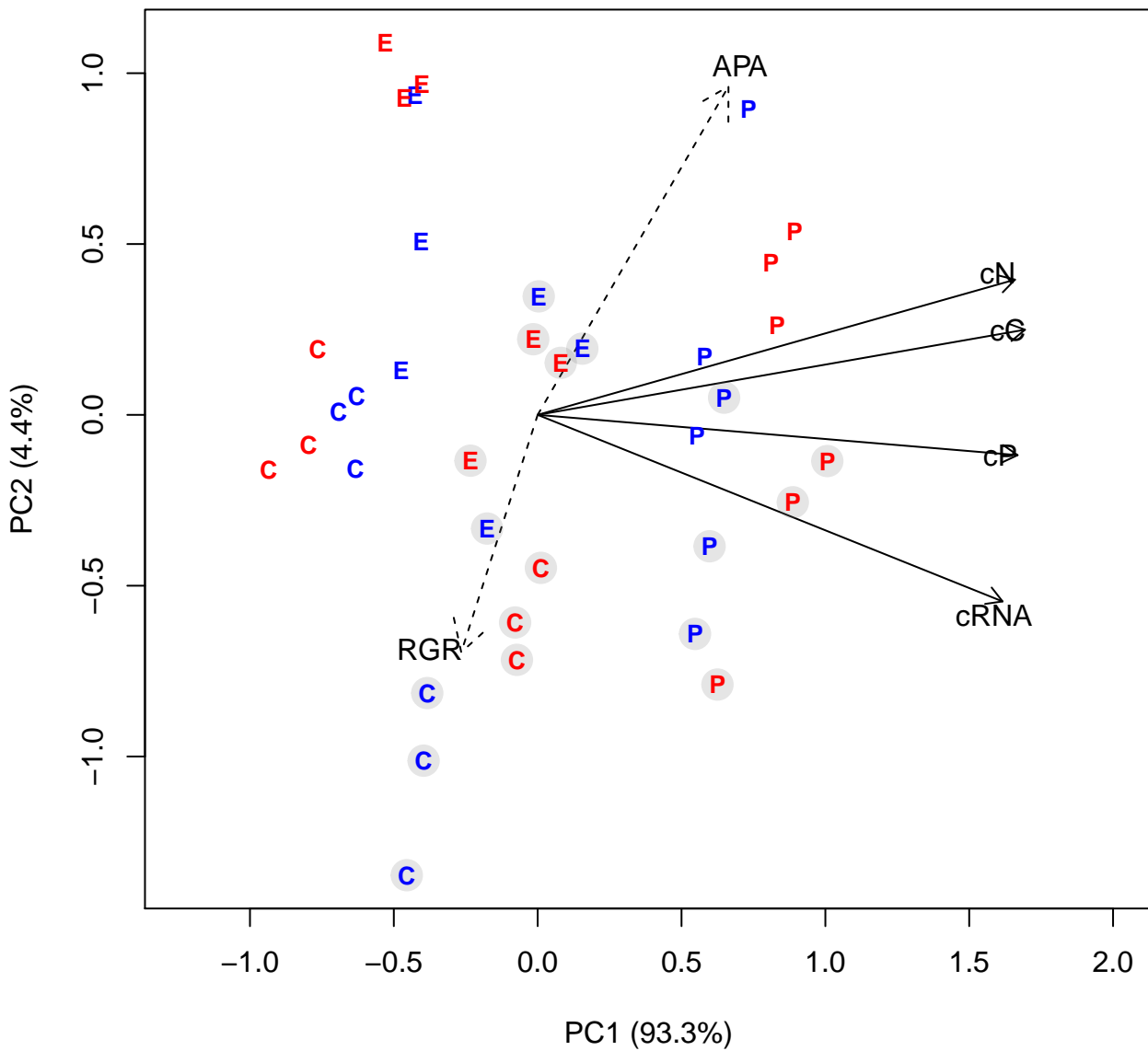

Supplement: Figure S2 — PCA plot based on log-transformed and standardized cell quota of C, N, P, and RNA in the 36 experimental units. Due to the factorial design consisting of three species, two temperatures, and two P-regimes, we obtained 12 unique experimental combinations which are represented in the diagram as follows: The letters E, P, and C represents the species Emiliania huxleyii, Prymnesium polylepis, and Chrysochromulina rotalis; the red and blue color represents 19 °C and 13 °C, respectively; and the grey circles represent the +P cultures. PC axis 1 accounted for 93.3% of the variation in the dataset, while PC axis 2 accounted for 4.4%. Specific growth rate (μ) and alkaline phosphatase activity (APA) were included as passive variables in the PCA plot (they did not influence the ordination, but are fitted to the ordination afterwards; see methods) and plotted as dotted arrows. The arrows points in the direction to which the linear change in the variable is the fastest. Both were significantly correlated to the ordination (R2 for APA: 0.47, p < 0.01; R2 for growth rate: 0.19, p = 0.03). [file peerj-05-3743-s003.pdf]

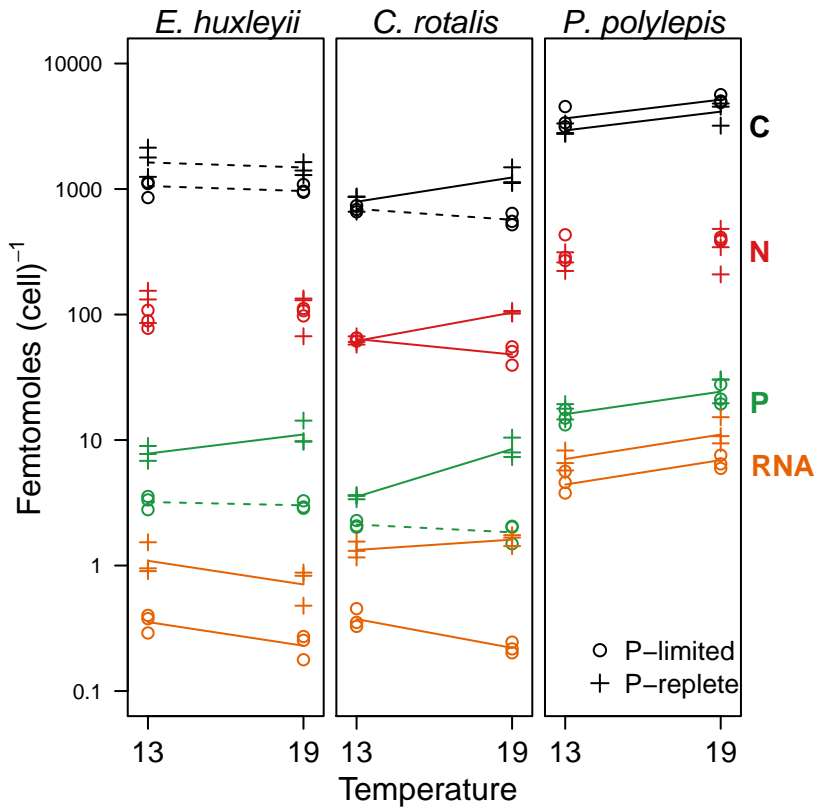

Supplement: Figure S3 — Responses of cell quota of C, N, P, RNA, C:P and N:P to temperature and P-regime. The data are plotted on log-scale. Dots represent P-limited cultures (−P), pluses P-replete cultures (+P). Solid lines are drawn if the difference between temperatures was significantly different from zero within a given P-regime (see Table 2 for coefficient estimates and p-values). Dotted lines indicate non-significant trends. If two lines are drawn for an element or a ratio, there was a significant difference between P-regimes for at least one of the two temperatures. If no line is drawn (as for N in P. polylepis), no P-regime- nor temperature-effect was present. [file peerj-05-3743-s004.pdf]

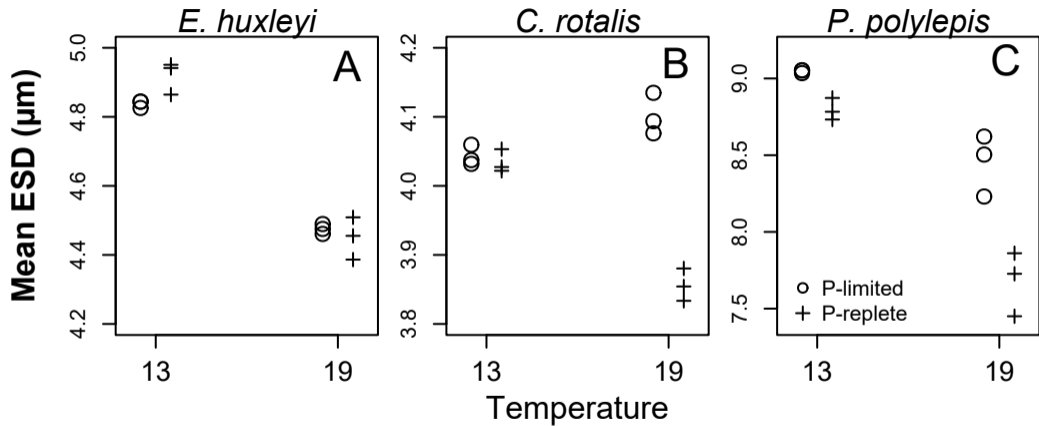

Supplement: Figure S4 — Mean cell size, calculated as the average equivalent spherical diameter (ESD) from weekly samplings over the >20-week experimental period, in the different treatments and species. Dots represent P-limited cultures (−P), pluses P-replete cultures (+P). [file peerj-05-3743-s005.pdf]
