# Supplementary material for: Growth, stoichiometry and cell size; temperature and nutrient responses in haptophytes
Source: PeerJ. 2017 Sep 5;5:e3743. doi: 10.7717/peerj.3743 (PMC5590550; doi:10.7717/peerj.3743)
Supplement: Table S1 — ANOVA table showing the sum of squares, fraction of variance explained, and p-value for each of the explanatory terms in a linear model with APA as the response variable. [file peerj-05-3743-s006.docx]

| Term | Sum sq | % of var | P |
| --- | --- | --- | --- |
| P-regime | 23.6905 | 58.7 | <0.0001 |
| temperature | 0.7662 | 1.89 | 0.078 |
| Species | 2.8570 | 7.1 | 0.006 |
| P-regime:species | 5.0600 | 12.5 | 0.0003 |
| temperature:species | 1.7540 | 4.3 | 0.035 |
| Residuals | 6.2032 | 15.4 | - |

**Table S1**: ANOVA table showing the sum of squares, fraction of variance explained, and p-value for each of the explanatory terms in a linear model with APA as the response variable.
