# Supplementary material for: Growth, stoichiometry and cell size; temperature and nutrient responses in haptophytes
Source: PeerJ. 2017 Sep 5;5:e3743. doi: 10.7717/peerj.3743 (PMC5590550; doi:10.7717/peerj.3743)
Supplement: Table S2 — PERMANOVA table for the variance in cell quota of C, N, P and RNA that was related to species, P-regime, temperature, and the interaction between temperature and P-regime. The % of variance (var) is calculated from the sum of squares (Sum sq) of each term divided by the total sum of squares. [file peerj-05-3743-s007.docx]

**Table S2:** PERMANOVA table for the variance in cell quota of C, N, P and RNA that was related to species, P-regime, temperature, and the interaction between temperatura and P-regime. The % of variance (var) is calculated from the sum of squares (Sum sq) of each term divided by the total sum of squares.

| Term | Sum sq | % of var | P |
| --- | --- | --- | --- |
| Species | 4.4 | 62.0 | 0.001 |
| P-regime | 1.11 | 15.7 | 0.001 |
| temperature | 0.15 | 2 | 0.057 |
| P-regime:temperature | 0.049 | 0.6 | 0.35 |
| Residuals | 0.046 |  | - |
