# Supplementary material for: Growth, stoichiometry and cell size; temperature and nutrient responses in haptophytes
Source: PeerJ. 2017 Sep 5;5:e3743. doi: 10.7717/peerj.3743 (PMC5590550; doi:10.7717/peerj.3743)
Supplement: Table S3 — Results from linear models of cell quota of C, N, P and RNA. Legend as in Table 2 in manuscript. [file peerj-05-3743-s008.docx]

|  | *β*_0_ (Intercept) | *β*_1_ (P-regime) | *β*_2_ (Temp) | *β*_3_  (P-regime : Temp) | R^2^ (% explained by each variable) |
| --- | --- | --- | --- | --- | --- |
| *E.huxleyii*  C  N  P  RNA | -11.97 ***  -12.98 ***  -14.49 ***  -15.45 *** | 0.188*  NS  0.386***  0.488*** | -0.04 (NS)  NS  -0.026 (NS)  -0.189* | NS  NS  0.179*  NS | 0.67 (67, -,-)  -  0.95 (91, 2, 3)  0.89 (77, 12, -) |
| *C. rotalis*  C  N  P  RNA | -12.16 ***  -13.2 ***  -14.67 ***  -15.42 *** | 0.057 (NS)  -0.015 (NS)  0.22*  0.55*** | -0.086 (NS)  -0.12*  -0.06 (NS)  -0.23* | 0.279*  0.349***  0.44**  0.31* | 0.88 (56, 4, 28)  0.92 (40, 4, 48)  0.97 (70, 9, 18)  0.98 (93, 1,5) |
| *P. polylepis*  C  N  P  RNA | -11.43 ***  -12.49 ***  -13.79 ***  -14.35 *** | -0.96*  NS  NS  0.204* | 0.149*  NS  0.179*  0.195* | NS  NS  NS  NS | 0.71 (50, 21)  -  0.6 (60, -,-)  0.8 (42,38) |

**Table S3:** Results from liner models of cell quota of C, N, P and RNA. Legend as in table 2 in manuscript.

*** < 0.0001, ** < 0.001, * < 0.05
