# Supplementary material for: Growth, stoichiometry and cell size; temperature and nutrient responses in haptophytes
Source: PeerJ. 2017 Sep 5;5:e3743. doi: 10.7717/peerj.3743 (PMC5590550; doi:10.7717/peerj.3743)
Supplement: Table S4 — ANOVA tables showing the sum of squares (Sum sq), fractions of variance explained (% var), and p-values for the explanatory terms (P-regime, temperature (Temp) and their interaction) in a linear model with mean cell size as the response variable. ANOVAs were done separately for each specie to remove the large variance in cell size between species. [file peerj-05-3743-s009.docx]

| Species | Term | Sum sq | % of var | p |
| --- | --- | --- | --- | --- |
| *E. huxleyi* | P-regime | 0.00235 | 0.4 | 0.25 |
|  | Temp | 0.518 | 95.7 | < 0.001 |
|  | P:temp | 0.0084 | 1.6 | 0.05 |
|  | Residuals | 0.00158 | 2.3 | - |
| *C. rotalis* | P-regime | 0.0485 | 46.1 | < 0.001 |
|  | Temp | 0.011 | 10.2 | <0.01 |
|  | P:temp | 0.042 | 40 | < 0.001 |
|  | Residuals | 0.00049 | 3.7 | - |
| *P. polylepis* | P-regime | 0.785 | 23.3 | < 0.001 |
|  | Temp | 2.198 | 65.3 | < 0.001 |
|  | P:temp | 0.204 | 6.1 | < 0.05 |
|  | Residuals | 0.0223 | 5.3 | - |

**Table S4**: ANOVA tables showing the sum of squares (Sum sq), fractions of variance explained (% var), and p-values for the explanatory terms (P-regime, temperature (Temp) and their interaction) in a linear model with mean cell size as the response variable. ANOVAs were done separately for each specie to remove the large variance in cell size between species.
